# Structure and Function of Trypsin-Loaded Fibrinolytic Liposomes

**DOI:** 10.1155/2017/5130495

**Published:** 2017-07-03

**Authors:** Anna Tanka-Salamon, Attila Bóta, András Wacha, Judith Mihály, Miklós Lovas, Krasimir Kolev

**Affiliations:** ^1^Department of Medical Biochemistry, Semmelweis University, Budapest, Hungary; ^2^IMEC, Research Centre for Natural Sciences, Hungarian Academy of Sciences, Budapest, Hungary

## Abstract

Protease encapsulation and its targeted release in thrombi may contribute to the reduction of haemorrhagic complications of thrombolysis. We aimed to prepare sterically stabilized trypsin-loaded liposomes (SSL_T_) and characterize their structure and fibrinolytic efficiency. Hydrogenated soybean phosphatidylcholine-based SSL_T_ were prepared and their structure was studied by transmission electron microscopy combined with freeze fracture (FF-TEM), Fourier transform infrared spectroscopy (FT-IR), and small-angle X-ray scattering (SAXS). Fibrinolytic activity was examined at 45, 37, or 24°C on fibrin or plasma clots with turbidimetric and permeation-driven lysis assays. Trypsin was shown to be attached to the inner surface of vesicles (SAXS and FF-TEM) close to the lipid hydrophilic/hydrophobic interface (FT-IR). The thermosensitivity of SSL_T_ was evidenced by enhanced fibrinolysis at 45°C: time to reduce the maximal turbidity to 20% decreased by 8.6% compared to 37°C and fibrin degradation product concentration in the permeation lysis assay was 2-fold to 5-fold higher than that at 24°C. SSL_T_ exerted its fibrinolytic action on fibrin clots under both static and dynamic conditions, whereas plasma clot dissolution was observed only in the permeation-driven assay. The improved fibrinolytic efficiency of SSL_T_ under dynamic conditions suggests that they may serve as a novel therapeutic candidate for dissolution of intravascular thrombi, which are typically exposed to permeation forces.

## 1. Introduction

Thrombolysis based on enzymatic dissolution of fibrin is currently the first-line treatment of ischemic stroke as well as certain selected cases of acute myocardial infarction [1, 2]. Most of the fibrinolytic agents are plasminogen activators, which can be classified as “indirect fibrinolytics,” because their enzymatic action is directed towards plasminogen, while, in contrast, fibrinolytics such as plasmin and its derivatives degrade fibrin without any intermediate step of plasminogen activation and are therefore designated as “direct fibrinolytics.” Systemic (intravenous) administration of indirect fibrinolytics is accompanied by frequent bleeding side effects related to the large excess of activator at its therapeutic dose over the inhibitor capacity of blood plasma [3]. In contrast, locally administered plasmin exerts its fibrinolytic action in fibrin-bound form being protected from its main inhibitor (*α*_2_-plasmin inhibitor) but is immediately inactivated when entering the circulation, thus preventing bleeding at remote sites of vascular injury (reviewed in [4]). However, catheter directed thrombolysis combined with systemic thrombolysis may be associated with higher risk of pulmonary embolism or intracranial haemorrhage compared to systemic thrombolysis alone [5].

Nanomedicine offers approaches for noninvasive and rapid thrombolytic treatment, with the hope of further reducing the morbidity and mortality of occlusive cardiovascular events. Being encapsulated into liposomes, drugs are preserved from metabolization prior to reaching target tissues, and simultaneously they minimize exposure of healthy tissue to the encapsulated drug during its circulation in the blood. The possibility to target liposomes helps in localizing sufficient quantities of thrombolytic agents to the desired thrombus. In vivo results show strong evidence that external targeting is superior to passive targeting of highly stable long-circulating drug formulations. A promising alternative for external targeting is achieved by temperature-triggered, localized intravascular drug release from thermosensitive liposomes with focused heating ([6], reviewed in [7]).

Trypsin could be a novel candidate for being a liposome-encapsulated thrombolytic drug because of its high fibrinolytic efficiency [8] and about 3.5 times lower molecular mass compared to that of plasmin allowing higher encapsulated enzyme concentration. If administered in a plasma environment, encapsulated trypsin is protected against plasma inhibitors, the most abundant of which is *α*_1_-PI (*α*_1_-protease inhibitor). However, in contrast to plasmin and tPA, trypsin lacks any structural domains for recognition and specific binding to fibrin. Thus, an alternative targeting strategy is required to allow for a local proteolytic action of trypsin in thrombi. Such an option is offered by the temperature-dependent release of the enzyme from the thermosensitive liposomes developed and characterized in the current study, which in vivo could be achieved with focused ultrasound thermal effects. Following the local release of trypsin in thrombi, the availability of the fibrin substrate will protect the enzyme from inactivation by *α*_1_-PI, similarly to the fibrin-mediated protection of other proteases (plasmin and PMN-elastase) against plasma protease inhibitors [9].

The purpose of our work was to prepare trypsin-loaded PEGylated liposomes and characterize them in terms of their structure and proteolytic efficiency in pure fibrin, as well as in plasma environment.

## 2. Materials and Methods

### 2.1. Proteins and Reagents

If not otherwise indicated, experiments were performed in HEPES buffered saline (HBS, 10 mM HEPES, 150 mM NaCl, pH 7.4). Porcine trypsin and bovine thrombin were purchased from Serva Electrophoresis GmbH (Heidelberg, Germany) and the latter was further purified as described in [10] yielding a preparation with specific activity of 2100 IU/mg. Thrombin activity of 1 IU/mL was considered equivalent to approximately 10.7 nM by active site titration [11]. Fibrinogen (human, plasminogen-depleted) was from Calbiochem (San Diego, CA, USA). Hydrogenated soybean phosphatidylcholine, cholesterol, distearoyl-phosphatidylethanolamine PEG2000, and cholesterol sulphate were acquired from Avanti (Birmingham, AL, USA). DPH (1,6-diphenyl-1,3,5-hexatriene) and fluorescamine were from Sigma-Aldrich Kft. (Budapest, Hungary) and Spectrozyme-PL (H-D norleucylhexahydrotyrosyl-lysine-p-nitroanilide) was from Sekisui Diagnostics (Pfungstadt, Germany). Citrated, fresh frozen plasma (fibrinogen concentration: 2.35 g/l) was obtained from the Hungarian Blood Supply Service (Budapest, Hungary).

### 2.2. Preparation of Trypsin-Loaded Liposomes

Trypsin-loaded (SSL_T_) and empty (SSL) sterically stabilized liposomes consisting of hydrogenated soybean phosphatidylcholine, cholesterol, distearoyl phosphatidylethanolamine PEG2000, and cholesterol sulphate at a molar ratio of 15 : 4 : 1 : 1 were prepared by thin-layer evaporation method as follows. Constituents were mixed and dissolved in a chloroform/methanol mixture (95 : 5 volume ratio) at a total phospholipid concentration of 34 mg/ml. Evaporation of the organic solvent was facilitated by a vacuum pump (20 mbar, at 24°C, overnight). The lipid film was hydrated at room temperature with HBS for preparing empty liposomes or 0.1 mM HCl in distilled water containing 10 mg/ml porcine trypsin for trypsin-loaded liposomes and stirred for 10 min at 1200 rpm. Formation of unilamellar vesicles was promoted by sonication (three cycles of 30 s at 50 watt, 20 kHz in a Branson Sonifier 250, Branson Ultrasonics Corp., Danbury, CT, USA) followed by ten freeze-thaw cycles (−78°C; +42°C). SSL_T_ was then extruded through a 100 nm pore diameter polycarbonate filter in a LiposoFast mini-extruder (Avestin Inc., Ottawa, Canada). Nonencapsulated trypsin and lipid debris were removed from supernatant by centrifugation (3 times for 15 min at 133,000*g*) with a Beckman Airfuge 340401 ultracentrifuge. The sediment was resuspended in HBS yielding a liposome preparation of neutral pH, with trypsin encapsulated in its inactive form due to the low pH inside of the enzyme loaded vesicles. The concentration of phospholipids in the SSL_T_ suspension was determined with the fluorescent probe DPH [12] yielding a concentration of about 30 mg/ml. Changes in enzyme activity of the encapsulated trypsin were monitored daily on the chromogenic small peptide substrate Spectrozyme-PL (SpPL) at 405 nm after lysing liposomes by stirring together with 2.5 v/v% Triton X-100 for ten seconds at 1200 rpm. SSL_T_ was stored at 4°C until use.

### 2.3. Dynamic, Permeation-Driven Lysis of Fibrin and Plasma Clots

In order to examine the fibrinolytic activity of our liposome preparation under dynamic conditions, fibrin and plasma permeation studies were performed at 24 and 45°C. The inner surfaces of 5 ml pipette tips (Finntip, Thermo Scientific, Budapest, Hungary) were precoated with 1 g/l fibrinogen for 3 h and then air-dried [13]. Fibrin clots were prepared from fibrinogen at 7.5 *μ*M clotted with 16 nM thrombin in the fibrinogen-coated pipette tips. Plasma clots were prepared from citrated, fresh frozen plasma supplemented with fibrinogen to 7.5 *μ*M final concentration and CaCl_2_ to 12.5 mM and filled in the tips. After 70 min of incubation at 37°C, stable clots were washed thoroughly with HBS to remove unclotted fibrinogen and other plasma proteins. Trypsin-loaded liposomes (50 *μ*l) containing phospholipid at 5 mg/ml and enzyme activity corresponding to that of 300 nM free trypsin, empty liposomes, or HBS were layered over clots of 200 *μ*l volume. After the entry of the liposomal suspension into the clots, HBS was added and continuously supplemented to keep a constant hydrostatic pressure over the clots. The pipette tips were kept at 24 or 45°C during the lytic process. Consecutive fractions of 50 *μ*l were then collected and their protein concentration was determined by the fluorescamine method [14] and plotted against the eluted volume. Fractions with the highest protein content were analyzed with SDS polyacrylamide gel electrophoresis (SDS PAGE) in 4–15% gradient gels under nonreducing conditions, followed by visualization of protein bands with silver staining.

### 2.4. Lysis of Fibrin and Plasma Clots in a Turbidimetric Assay

Two different experimental setups were designed to measure the fibrinolytic effectiveness of our liposomes under static conditions. First, fibrinogen at 7.5 *μ*M or plasma supplemented with fibrinogen (7.5 *μ*M) and calcium (12.5 mM) were clotted with thrombin (20 nM) at 37°C in 96-well microtiter plates. Clot dissolution was started by layering 50 *μ*l of SSL_T_ (or empty liposomes or HBS) containing phospholipid at about 30 mg/ml and enzyme activity corresponding to that of 1.8 *μ*M trypsin over the clot (“extrinsic lysis” assay), followed by measuring the light absorbance at 340 nm at 37°C with a Zenyth 200rt microplate spectrophotometer (Anthos Labtec Instruments GmbH, Salzburg, Austria) and in parallel at 45°C with a CLARIOstar® microplate reader (BMG LABTECH GmbH, Ortenberg, Germany). In a second (“intrinsic lysis”) setup, fibrinogen and plasma clots were prepared as above, but prior clotting SSL_T_ (or SSL) was homogeneously dispersed in the clotting mixture at 3 mg/ml final phospholipid concentration and 180 nM trypsin. The clotting and the clot dissolution phases were monitored by measuring the light absorbance at 37°C and 45°C in parallel. The CLARIOstar® microplate reader was used in an orbital averaging mode. Using this mode, the measurement takes place on an orbit with definable diameter within each well. It results in higher absorbance values compared to the measurement using a Zenyth 200rt microplate spectrophotometer in a normal mode, when one point is measured in the middle of each well. Therefore, data gained at 37°C with the Zenyth microplate reader were multiplied by a factor of 2.41 to be comparable with those gained at 45°C with the other instrument. For comparison of lytic rates of the two static assays, the time needed to reduce the turbidity of the clots by a given fraction of the maximal value (*T*_20_, *T*_50_, and *T*_80_ to reach 0.8, 0.5, and 0.2*A*_max_, resp.) was calculated as a quantitative parameter of fibrinolytic activity. The curve analyzing process and statistical comparison of the *T* values with Kolmogorov-Smirnov test were performed in Matlab R2016a (The MathWorks, Inc., Natick, MA, USA).

### 2.5. Transmission Electron Microscopy Combined with Freeze Fracture (FF-TEM)

A 1-2 *μ*l droplet of SSL_T_ or SSL suspension was used for freeze fracturing. The samples were pipetted to the golden sample holder and rapidly frozen in the mixture of liquid and solid Freon, cooled by liquid nitrogen. Fracturing was performed at 173 K in a freeze fracture device (BAF 400D, Balzers AG, Liechtenstein). The fractured surfaces were etched for 30 s at 173 K and then shadowed by platinum and covered with carbon. The replicas obtained were washed with surfactant and water and were finally transferred to 200-mesh copper grids. The electron micrographs were made in a Philips Morgagni 268D electron microscope.

### 2.6. Fourier Transform Infrared Spectroscopy (FTIR)

For FTIR spectroscopic study, the attenuated total reflection (ATR) technique was used, having a penetration depth of infrared light in the samples in the order of one micrometer, so the investigation in bulk aqueous phase was possible, too.

ATR-FTIR spectroscopic measurements were carried out by means of a Varian 2000 (Scimitar Series) FTIR spectrometer fitted with a diamond attenuated total reflection cell (Specac's “Golden Gate” single reflection ATR unit with active area of 600 × 600 *μ*m^2^). SSL_T_ (or SSL) suspension (5 *μ*l) was spread onto the diamond ATR surface. Room temperature spectra (128 scans, resolution of 2 cm^−1^) were recorded both as suspension using a cap to avoid sample drying and as dry films after slow evaporation of the buffer solvent under ambient conditions. ATR correction was executed after each data collection. All spectral manipulations were performed using GRAMS/32 software package (Galactic Industries Incorporation, USA).

### 2.7. Small-Angle X-Ray Scattering (SAXS)

Small-angle X-ray scattering measurements were performed using CREDO, an in-house transmission geometry setup [15]. SSL_T_ and SSL samples (at 20 mg/ml phospholipid) were filled into thin-walled quartz capillaries of 1.2 mm average outer diameter. After proper sealing, these were placed in a temperature-controlled aluminium block, which was inserted into the vacuum space of the sample chamber. Measurements were done using monochromatized and collimated Cu K*α* radiation (0.1542 nm wavelength), and the scattering pattern was recorded in the range of 0.23–1.03 nm^−1^ in terms of the scattering variable (defined as *q* = (4*π*sin⁡*ϑ*)/*λ*, where 2*ϑ* is the scattering angle and *λ* is the X-ray wavelength). The total measurement times were 7.5 hours for each sample. In order to assess sample and instrument stability during the experiment, the exposures were made in 5-minute units, with frequent sample change and reference measurements. The same measurement protocol was used in our previous study executed on artificial nanoerythrosomes [16]. These individual exposures were corrected for beam flux, geometric effects, sample self-absorption, and instrumental background and calibrated into physical units of momentum transfer (*q*, nm^−1^) and differential scattering cross section (absolute intensity, cm^−1^ × sr^−1^). The averages of all the corrected and calibrated scattering patterns for each sample were azimuthally averaged to yield single one-dimensional scattering curves for each sample [17–19].

## 3. Results and Discussion

### 3.1. Evaluating the Fibrinolytic Function of SSL_T_

According to the trypsin activity measurements, enzyme activity of our SSL_T_ preparation on the first day (100%) was equivalent to the activity of 1.8 *μ*M free trypsin. Then, following an initial drop of 30% during the first 3 days, it remained unchanged for 10 days stored at 4°C (data not shown).

**Figure 1:**
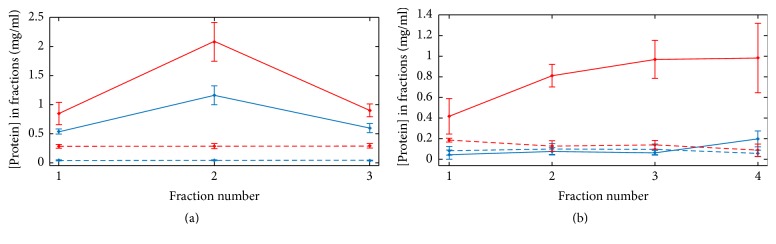
Permeation-driven lysis of fibrin and plasma clots. SSL_T_ (solid lines) or HBS (dashed lines) was layered to the surface of fibrin (a) or plasma (b) clots; thereafter a constant hydrostatic pressure was maintained over the clots and the eluted fluid was collected in fractions of 50 *μ*l each, in which the protein content was measured and is shown as mean ± SEM (*n* = 3). Red and blue colours indicate clot lysis at 45°C and 24°C, respectively.

The fibrinolytic activity of SSL_T_ was tested under dynamic conditions with permeation of fibrin and plasma clots at room temperature and 45°C in parallel to test the thermosensitivity of the liposome vehicle construct. Trypsin-loaded liposomes were layered over the surface and after their entry into the clots HBS was added and continuously supplemented to keep a constant hydrostatic pressure over the clots. The eluted fluid was collected in fractions and analyzed for protein content, indicating the release of soluble fibrin degradation products (Figure 1).

Elevated temperature during the lytic phase markedly enhanced the lytic efficiency of the trypsin-loaded liposomes. Close to the phase transition temperature enhanced area fluctuations and increased lateral compressibility of the liposome membrane lead to increased likelihood of spontaneous lipid pore formation and therefore to permeation events [20] and the consequent release of the encapsulated enzyme. Thus, increased lytic efficiency at elevated temperatures proves that the applied liposome is a* thermosensitive* construct. The upper limit of mild hyperthermia during cancer therapy is 45°C, while higher temperatures are used for ablation techniques causing serious damage in the tumor tissue, but cells with normal vascularization and normal heat-shock protection mechanisms might be safely subjected to a thermal treatment of 45°C [21, 22]. This local thermal effect could be achieved in thrombi with high intensity focused ultrasound with controlled and selected parameters. The conformal microwave array applicators are also potential hyperthermia applications that may be suitable for heat-assisted thrombolysis [22–25].

Nevertheless, (i) the drug release at 24°C, (ii) the slightly elevated level of detectable protein in fractions of the control sample at 45°C in a fibrin environment (Figure 1(a)), and (iii) the lower detectable protein concentrations in fractions collected during plasma permeation with SSL_T_ (Figure 1(b)) need further consideration.

(i) Since SSL_T_ was stored at 4°C before and during trypsin activity measurements, the observed stability of the encapsulated enzyme provides information about drug retention capacity at low temperatures. Therefore, it cannot be excluded that at 24°C protein leakage increases from SSL_T_ due to changes of the organization in the SSL_T_ double layer. Furthermore, despite the fact that PEGylation is the most common method for vesicle stabilization and preventing interactions between the particle surface and plasma proteins, interactions between PEGylated nanoparticles and albumin, fibrinogen, IgG, and apolipoproteins have been reported [26]. Moreover, interactions of antimicrobial [27], cytoskeletal [28, 29], and other proteins (calponin [30]) with lipid vesicles have been found to cause vesicle leakage. Since fibrinogen is known to bind phospholipids [31, 32], drug release from SSL_T_ due to interaction with fibrin(ogen) is also a plausible explanation for detectable fibrin degradation at 24°C during fibrin permeation by trypsin-loaded vesicles.

(ii) Mild disintegration at 45°C due to less stable structure of fibrin clots compared to that of plasma clots containing calcium and FXIII (Figure 1(b), red, dashed lines) may lead to the constant, slightly elevated level of detectable protein in fractions of the control sample in the case of fibrin clots (Figure 1(a), red, dashed lines).

(iii) Lower protein concentrations of the eluted fractions in the plasma clot permeation (Figure 1(b)) compared to the fibrin clot permeation (Figure 1(a)) are presumably the consequence of the plasma clot being more resistant to lysis compared to the fibrin clot due to the possibility to form cross-links during the clotting phase. Furthermore, previous studies have shown that *α*_1_-antitrypsin is noncovalently bound to fibrin clots prepared from plasma, preserving its serine protease inhibitor activity despite extensive washing [33]. The presence of such an effective inhibitor of trypsin may also lead to decreased fibrinolytic efficiency of SSL_T_ in plasma clots.

**Figure 2:**
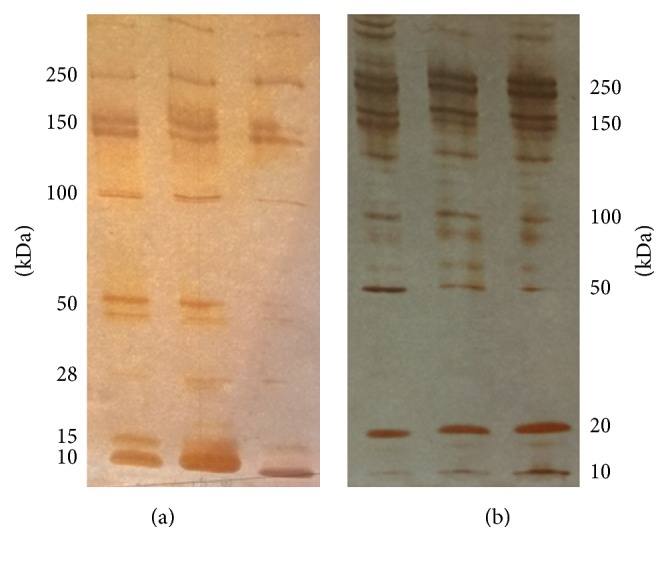
SDS polyacrylamide gel electrophoresis of protein fractions with the highest concentrations collected during fibrin and plasma clot permeation by SSL_T_. Three parallel fractions collected during fibrin (panel (a), fraction number 2) and plasma (panel (b), fraction number 3) clot permeation with SSL_T_.

SDS polyacrylamide gel electrophoresis was performed to examine the protein size distribution of the fractions with the highest protein concentrations in both cases (Figure 2).

A plasmin-like degradation pattern of fibrin [34] was observed with different types of fibrin degradation products, which evidenced that the protein content of fractions with the highest protein concentrations originated exclusively from fibrin degraded by trypsin. It should be noted that no fibrin degradation was found after layering empty liposomes over the fibrin or plasma clots.

**Figure 3:**
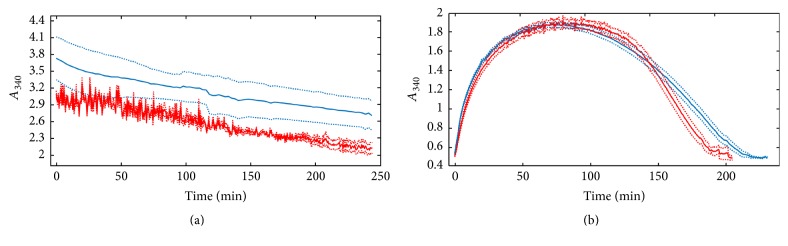
Lysis of fibrin by SSL_T_ layered on the clot surface or homogeneously dispersed in the clots. The liposomal solution was layered over preformed clots (a) or added to fibrinogen before the clotting phase (b) and thereafter formation and/or dissolution of fibrin were followed as changes in absorbance at 340 nm shown as mean (solid lines) ± SEM (dotted lines) of three measurements. Red and blue colours indicate clot lysis at 45°C and 37°C, respectively.

In addition to the permeation studies, the fibrinolytic activity of SSL_T_ was investigated under static conditions in “extrinsic lysis” assay by liposomes layered over and in “intrinsic lysis” assay by liposomes incorporated into fibrin and plasma clots at 37 and 45°C. In the extrinsic lysis setup, the complete dissolution of fibrin clot was achieved in 4 h. No significant difference was found between lysis times at the two different temperatures applied (Figure 3(a) and Table 1).

In the “intrinsic lysis” setup, SSL_T_ was incorporated into the clots during the clotting phase. Then, clots were let to dissolve on their own at different temperatures. The time needed for complete dissolution was about 2–2.5 hours at both examined temperatures, although a significant decrease in *T*_80_ (time needed to reduce the turbidity of the clots by 80% of *A*_max_) was observed at 45°C, implying faster trypsin release at the higher temperature again (Figure 3(b) and Table 1).

Under static conditions, there was no change in the absorbance values after the clotting phase in the control fibrin and plasma clots treated with HBS or SSL or in the plasma clots with HBS, SSL, or SSL_T_ (data not shown).

The failure to dissolve plasma clots under static conditions can be traced back to the effect of *α*_1_-antitrypsin retained in the clot. Furthermore, the SSL_T_ suspension was diluted to a greater extent in the intrinsic lysis setup compared to the permeation of plasma clots; thus the concentration of trypsin released from liposomes was not sufficiently high to counteract its inhibitor(s). Finally, the permeation pressure in the dynamic assay is an additional factor contributing to the enhanced release of the entrapped enzyme compared to the liposomes stationary incorporated into the clot. The heat-dependence of the trypsin release from SSL_T_ was much less pronounced and observed only in the late stage (*T*_80_, Table 1) of the static lysis assays, probably due to the smaller difference between the two temperatures applied.

The large difference in the time needed for total clot dissolution during surface (about 4 h), and inner (about 2 h) dissolution despite the tenfold liposome concentration in the former case is not unexpected. A similar difference is observed in the fibrinolytic potency of plasmin layered over fibrin and incorporated into fibrin clots; about a 100-fold higher enzyme concentration is needed to achieve a comparable lysis rate from the surface than with clot-entrapped plasmin [35, 36].

### 3.2. Structural Characterization of SSL_T_

**Figure 4:**
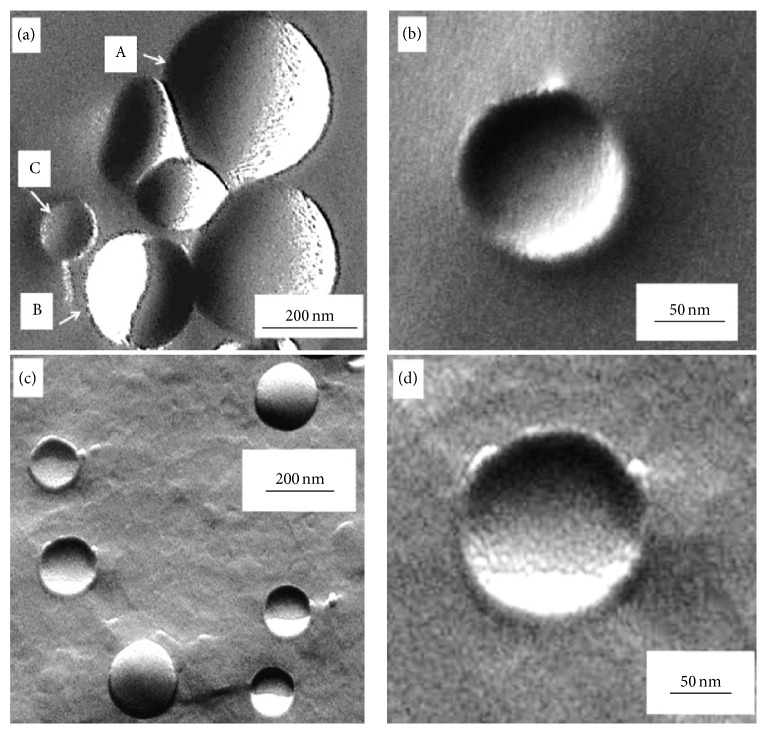
The surface morphology of the trypsin-free ((a) and (b)) and trypsin-loaded ((c) and (d)) vesicles by freeze fracture TEM. (a) Three types of fractures can be observed: (A) convex fractured surfaces of the vesicles protruding from the aqueous medium, (B) concave areas of the imprints of vesicles broken out, and (C) bottom parts of remaining vesicles broken through entirely. (b) The inner surface of a vesicle, broken through entirely, is typically smooth. All three types of fractured surfaces are observed in the trypsin-loaded vesicles (c), but the inner surface contains also closely packed grains (d).

The method of TEM combined with freeze fracture provides an excellent tool to visualize the internal structure of vesicle systems of sizes spanning from nanometers up to several micrometers [37]. The typical fractured surfaces of the nearly spherical vesicles are shown on Figure 4(a). Apart from the convex fractured surfaces of the vesicles protruding from their flat neighbourhood (“A”) (corresponding to the original, aqueous medium) we can observe the concave imprints of vesicles broken out entirely from the medium (“B”). These two types of fractured creations feature sharp contours. The third type of characteristic fractured surfaces represents vesicles that are broken through (“C”), leaving their bottom parts in the medium. Instead of being sharp, the contours of these are rather wide, corresponding to the wall thickness of the vesicles.

Taking a closer look at the electron micrographs, we can see that the outer surfaces of the vesicles are smooth (Figure 4(a)). The limited roughness of these areas, and also that of the inner surface of the remaining vesicle part, is the consequence of the platinum grains formed during the shadowing procedure, with a mean size of approx. 1-2 nm (Figure 4(b)).

In the trypsin-containing vesicles, a similar surface pattern can be observed in the outer convex and concave surfaces (Figure 4(c)), but a distinct morphological feature emerges in the third type of fracturing. The fractured surface morphology of the loaded vesicles exhibits a bit higher surface roughness: in the matrix around the vesicles the grain size is about 2 ± 1 nm, while inside it is about 4 ± 1 nm as seen in higher magnification image (Figure 4(d)). This observation indicates that the trypsin molecules are attached to the inner surface of the vesicles and are presumably not dispersed in their inner/aqueous core.

**Figure 5:**
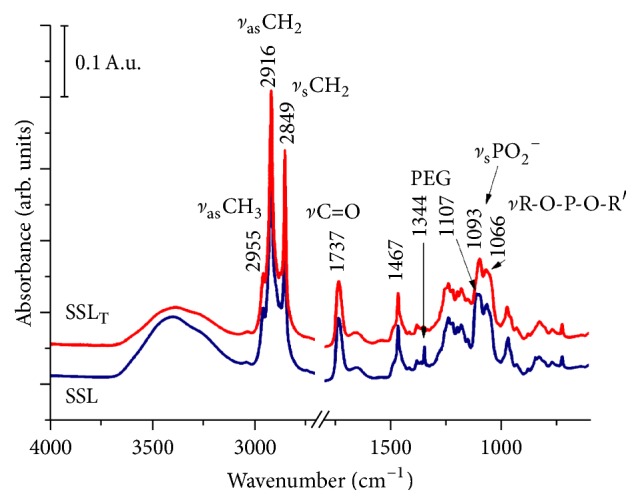
Dry film spectra of trypsin-loaded (SSL_T_) and empty (SSL) liposomes.

IR spectroscopy is suitable to reveal possible enzyme-lipid interaction by analysis of small spectral changes (band position and intensity) of both lipid- and enzyme-related IR bands.  Figure 5 shows the ATR-FTIR spectra of empty and trypsin-loaded liposomes recorded as dry films (after slow evaporation of buffer solvent on the top of the diamond ATR element).

**Figure 6:**
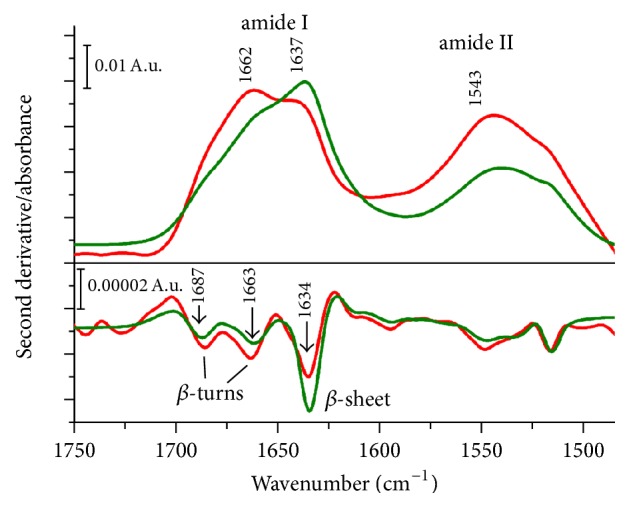
Amide I spectral region of trypsin before (green) and after (red) encapsulation in liposomes. The second derivatives of the measured spectra were obtained by the Savitzky-Golay method (3rd grade polynomial, 5 smoothing points).

Subtracting the spectra reveals the presence of amide I and amide II bands around 1653 and 1547 cm^−1^, respectively, corresponding to the presence of trypsin in trypsin-loaded liposomes (Figure 6, SSL_T_ spectrum). FTIR spectroscopy is widely applied to study protein secondary structure and aggregation. In particular, the amide I band (1700–1600 cm^−1^), corresponding mainly to the C=O stretching vibration of the peptide bond (~80%), is sensitive to the protein backbone conformational changes. Figure 6 shows the amide I spectral region of trypsin spectra before (in solution) and after encapsulation in liposomes. Trypsin is classified as a “*β*-protein” with dominant content of *β*-sheet and *β*-turn conformations [38]. After encapsulation, the relative amount of *β*-sheet conformers is slightly decreased, while the band component corresponding to turns and *β*-turns structure is slightly increased, reflected also in the shape of the amide I band envelope.

To get information about the spectral changes linked to the lipid molecules/bilayer, spectra recorded as suspension were analyzed after buffer subtraction. The C-H stretching region of the IR spectra (3020–2800 cm^−1^) involved mostly the fatty acyl chains of the lipids (not shown). No differences were observed for trypsin-loaded and empty liposomes, suggesting that the order/disorder of the acyl chains remained intact. Concerning the lipid head group spectral region (1800–900 cm^−1^), no changes in the phosphate vibrations (*ν*asPO_2_^−^ at 1234 cm^−1^, *ν*sPO_2_^−^ at 1088 cm^−1^, and *ν*R-O-P-O-R′ around 1070 cm^−1^) and in the antisymmetric C-N^+^ stretching vibrations (971 cm^−1^) of the choline groups could be revealed.

**Figure 7:**
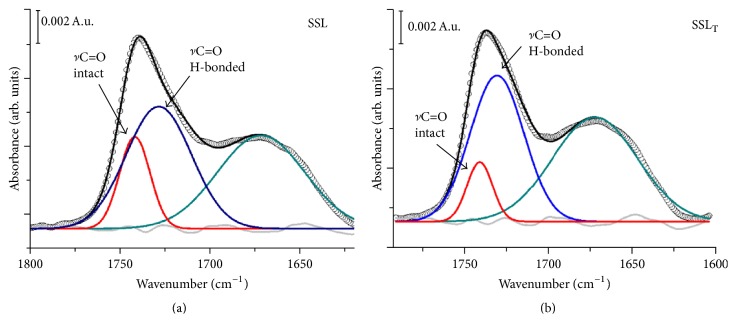
Lipid ester carbonyl stretching band region for empty (SSL) and trypsin-loaded (SSL_T_) liposomes. The solid black lines are the measured spectra; the blue and the red lines are the fitted bands corresponding to intact and H-bonded C=O groups, respectively. The green band component can be attributed to residual water (after subtraction) and to amide I band positions for curve fitting using the second derivative; band shapes were approximated by Lorentzian functions. The intensities and the bandwidth of each component were optimized according to a *χ*^2^ minimization procedure. The relative contribution of each of the particular components was calculated from their integrated areas in the best fit.

The lipid carbonyl ester group is situated in the interfacial region of the lipid bilayer and is a potential H-bond formation site. In highly hydrated phospholipid bilayers, this band splits in two overlapping components: a high wavenumber band around 1742 cm^−1^ of the non-hydrogen-bonded C=O groups and a low wavenumber band around 1728 cm^−1^ due to the hydrogen bonding of the C=O groups [39]. Detailed analysis of the *ν*C=O band around 1737 cm^−1^ (Figure 7) indicated an increase of the relative amount of H-bonded C=O subpopulation: *A* (*ν*C=O H-bonded)/*A* (*ν*C=O free) from 2.5 for SSL to 5.2 for SSL_T_. These data suggested that trypsin was located close to the lipid hydrophilic/hydrophobic interface driven by weak interaction (H-bonds).

In summary, the IR spectroscopic data evidence that the presence of trypsin molecules does not distort the acyl chain order of the lipid bilayers composing the liposomes despite the weak interaction-driven (H-bonds) location of the encapsulated trypsin close to the lipid hydrophilic/hydrophobic interface.

**Figure 8:**
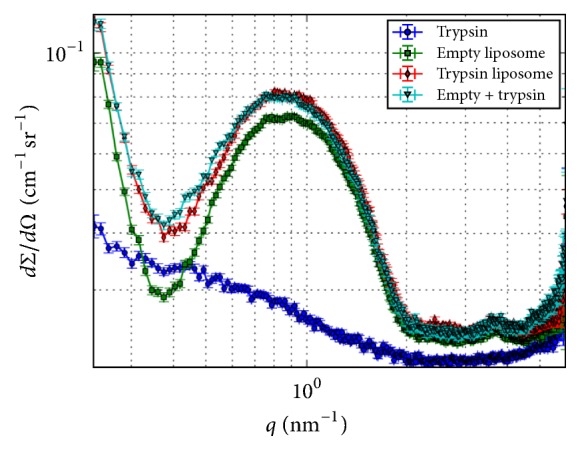
The one-dimensional scattering patterns of liposomes with (red) and without (green) trypsin. The scattering of trypsin in free solution (blue) and the curve generated as a sum of the scattering of empty liposomes and solved trypsin (cyan) are also shown.

Scattering techniques, especially small-angle X-ray scattering (SAXS), are a powerful tool for identifying various structural forms on the nanometer scale. To follow the trypsin-induced changes in the layer structure of the liposomes, SAXS measurements were performed. The one-dimensional scattering patterns of the empty and trypsin-loaded vesicles are shown in Figure 8. The scattering in the initial range of the variable is intense and decreases monotonously in both systems. This pattern originates from the scattering of domains spanning a size range of several, up to hundreds of nanometers. The two vesicle systems exhibit a similar scattering pattern with a broad peak in the *q*-range from 0.4 to 2 nm^−1^, which can be attributed to the scattering of the lipid bilayers, known as a form factor of the bilayer. However, there is a slight but important difference between the curves: the local minimum of the trypsin-containing vesicle is not as deep as in the case of empty vesicles.

The scattering of trypsin in its solvated form shows a monotonously decreasing SAXS pattern that is typically seen in the scattering of uncorrelated, nearly globular particles and corresponds to the nearly spherical shape of the trypsin molecule with a 3 nm diameter (Figure 8).

The SAXS curve of the trypsin-containing vesicle can be interpreted to the first approximation as the sum of the contributions of the empty vesicle and the trypsin. There is a slight difference, however, between the generated composite and the measured scattering of SSL_T_ curves (Figure 8). The difference is significant in the regime of the form factor, indicating the change in the bilayer structure and the association between the bilayer and the protein.

The scattering pattern of SSL_T_ can be fully reconstructed by a spherical shell model, enabling an approximate description of the layer structure (see Supplementary Material available online at https://doi.org/10.1155/2017/5130495). The model for the least-squares fitting of the bilayer scattering involves two identical, symmetrically placed Gaussian functions for the two head group regions, one Gaussian for the carbon chain region and two independent Gaussians for the guest molecules on the inner and outer side of the bilayer. The size distribution of the vesicles is assumed to be Gaussian.

The best fit (Supplementary Material) yields an asymmetric distribution in the head region of vesicle bilayer; therefore we may conclude that the protein molecules localize close to the head region. The thickness of the inner region of guest molecules (rich in trypsin) can be approximated by the sum of the radial displacement of the maximum of the corresponding Gaussian and its half width at half maximum (HWHM) which is 3.73 nm + 1.74 nm ≈ 5.5 nm. The SAXS fit has been executed without any a priori assumptions and the results are in agreement with those obtained by IR, namely, the fact that trypsin is located at the inner leaflet of bilayer in the vicinity of the lipid head group.

## 4. Conclusions

We have developed a phospholipid-based thermosensitive nanocarrier, in which trypsin is attached to the inner leaflet of the bilayer shell of the liposome. Our findings also represent a unique physicochemical description of a protein encapsulated in a lipid vesicle and are important in order to appreciate that the enzyme action depends on heat-dependent release and not simply on attachment to the outer surface of the vesicle. The fibrinolytic efficiency of these liposomes is improved in the dynamic fibrinolytic assay under conditions of permeation-driven fibrinolysis. Because intravascular thrombi are exposed to permeation forces [40, 41], these properties of our construct suggest that it could be a successful candidate as a therapeutic tool, the utility of which deserves further investigation.

## Supplementary Material

Modelling of liposome structure based on SAXS measurements.

## Figures and Tables

**Table 1:** Lysis times needed to dissolve fibrin clots by 20, 50, or 80%. Mean values ± SEM are shown (*n* = 3). Asterisk indicates statistical significance between the *T*_80_ values measured at the two temperatures at *p* < 0.05 according to Kolmogorov-Smirnov test.

	*T*_20_ (min)	*T*_50_ (min)	*T*_80_ (min)
Extrinsic lysis			
37°C	21.23 ± 6.96	89.75 ± 13.50	190.16 ± 9.02
45°C	42.99 ± 6.64	72.77 ± 14.71	167.71 ± 17.80
Intrinsic lysis			
37°C	131.44 ± 4.39	165.80 ± 4.70	192.24 ± 5.06
45°C	133.71 ± 1.15	156.41 ± 2.08	175.78^*∗*^ ± 2.88
