# Supplementary material for: Structure and Function of Trypsin-Loaded Fibrinolytic Liposomes
Source: Biomed Res Int. 2017 Jul 3;2017:5130495. doi: 10.1155/2017/5130495 (PMC5512056; doi:10.1155/2017/5130495)
Supplement: Supplementary file 1 — Modelling of liposome structure based on SAXS measurements. [file 5130495.f1.pdf]

Supplemental material

Structure and Function of Trypsin-loaded Fibrinolytic Liposomes

Anna Tanka-Salamon, Attila Bóta, András Wacha, Judith Mihály, Miklós Lovas, Krasimir Kolev

Small angle X-ray scattering (SAXS) for identifying the trypsin-induced changes in the layer structure of the liposomes

SAXS measurements were performed as described in the main text of the paper. This supplement describes the model of liposome structure generated from these data.

The model for the least-squares fitting of the bilayer scattering involves two identical, symmetrically placed Gaussian functions for the two head group regions, one Gaussian for the carbon chain region and two independent Gaussians for the guest molecules on the inner and outer side of the bilayer. The size distribution of the vesicles is assumed to be Gaussian.

The fit is shown in the next figure (labels as in Fig. 8 of the paper).

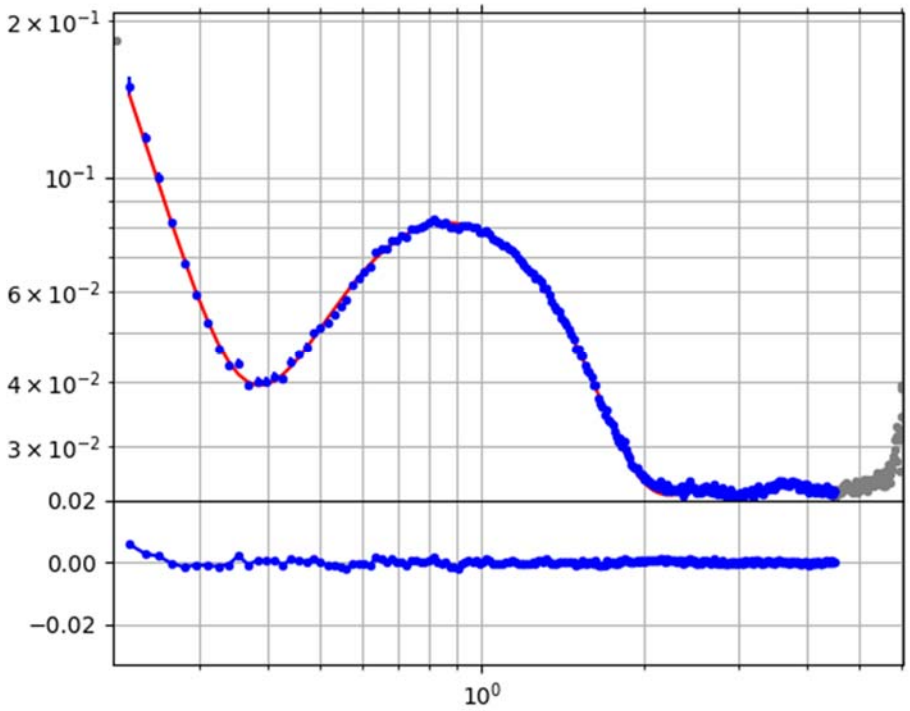

The data used for fitting are shown in blue. The end of the scattering curve (marked with gray) is not taken into account because of the noise due to the low scattering signal at the corner of the detector. The fitted model curve is shown in red. The residuals (difference of measured data and fitted values) indicating the goodness-of-fit are presented in the lower blue curve.

The parameters corresponding to the best fit are as follows.

Scaling factor: 1.3677517833084926·10-5
background: 0.0241995423701859
mean-radius of vesicle: 54.3 nm, σ=19.2569 nm
relative electron density of the carbon chain region: -1 (fixed by the model)
relative electron density of the inner guest region: 0.13084+/-0.06733

relative electron density of head group region: 1.09364+/-0.0937
relative electron density of the outer guest region: 0.02199+/-0.05379
distance of the inner guest region from the bilayer centre: 3.7285+/- 0.5171 nm
distance of the heads from the bilayer centre: 2.40537+/- 0.0103041 nm
distance of outer guest region from the bilayer centre: 0 ($5.2987 \cdot 10^{-24}$) nm
HWHM of the Gaussian for the inner guest region: 1.739516 +/- 0.348332 nm
HWHM of the Gaussian for the head group region: 0.227891 +/- 0.032714 nm
HWHM of the Gaussian for the carbon chain region: 0.4046934 +/- 0.038894955 nm
HWHM of the Gaussian for the outer guest region: 3.46557461 +/- 1.44277158 nm

The relative scattering length density (relative electron density taking into account the sigma values of the regions) is shown in the next figure.

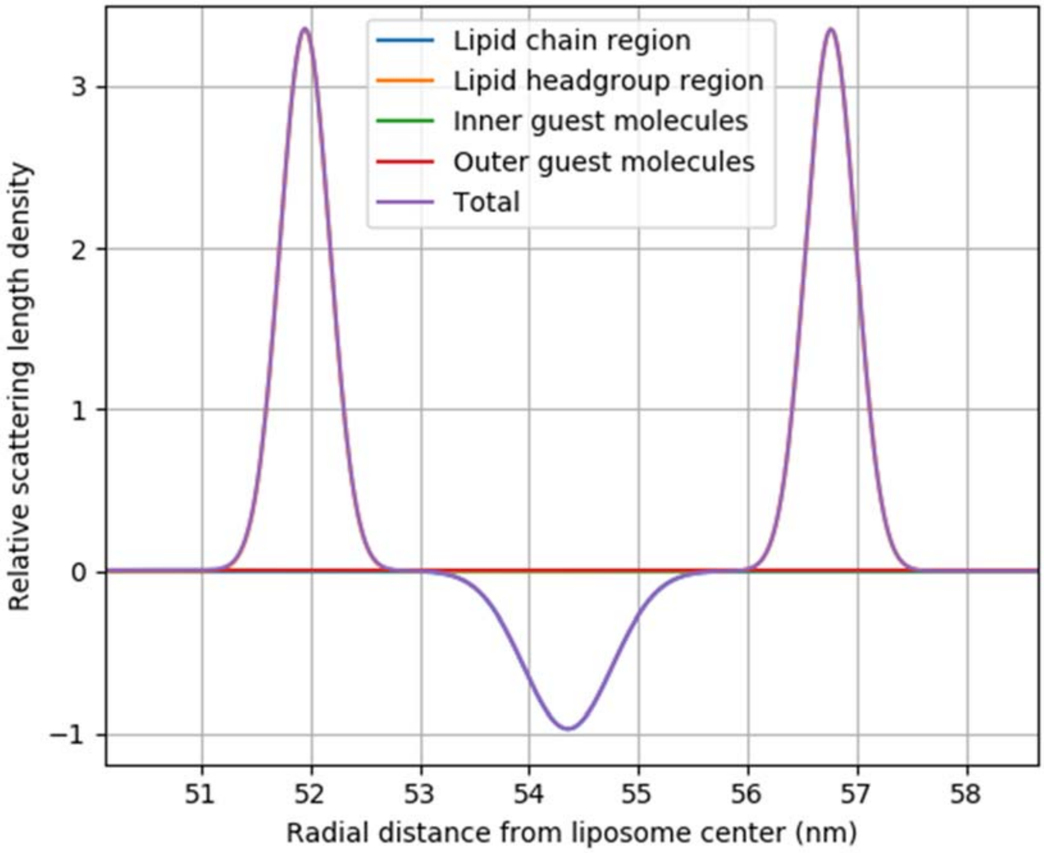

Although the guest regions exhibit significant electron contrast, their effects are minor in the scattering length because they are spread in a wide radial range. The high scattering length of the head regions originates from the cholesterol sulphate present in significant amount in the sample. After magnification we can observe an asymmetric distribution of the guest molecules as it is shown in the following figure. The inner guest region could be determined at a statistically significant level, contrary to the outer one, which has an almost negligible contribution.

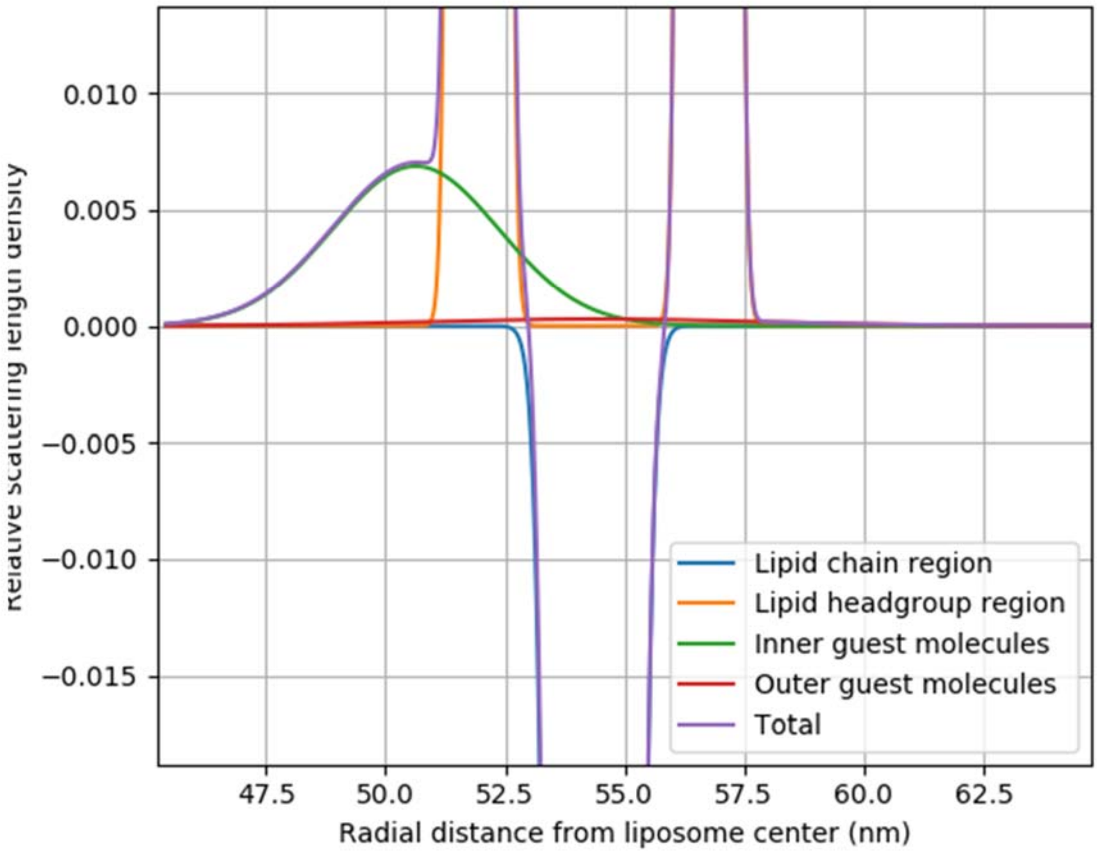

It is worth to mention that we have studied the radial distribution of sterically stabilized vesicles thoroughly [1,2]. In these cases an asymmetrical localisation of the PEGylated lipids was observed, meaning that the outer leaflet always contained PEG molecules in higher amount than the inner leaflet. In the present case, presumably the high electron contrast of cholesterol sulphate overshadows the much lower scattering power of the PEG chains. The higher contrast of the inner guest molecule region can consequently be attributed to trypsin molecules, thus this shell characterizes their location. The thickness of the inner region of guest molecules can be approximated by the sum of the radial displacement of the maximum of the corresponding Gaussian and its HWHM which is: 3.73 nm + 1.74 nm ≈ 5.5 nm. As the outer shell of guest molecules can be neglected we will omit its value in the manuscript. We agree with the reviewer that IR can carry solid data therefore those must be taken into account. The SAXS fit has been executed without any a priori assumptions and the result are in agreement with those obtained by IR, namely that trypsin is connected to the phospholipid head groups.

References

1. Z. Varga, Sz. Berényi, B. Szokol, L. Őrfi, G. Kéri, I. Peták, A. Hoell, and A. Bóta. A Closer Look at the Structure of Sterically Stabilized Liposomes: A Small-Angle X-ray Scattering Study**.** The journal of physical chemistry. B, vol. 114, pp. 6850–6854, 2010.
2. Z. Varga, A. Wacha, U. Vainio, J. Gummel and A. Bóta: Characterization of the PEG layer of sterically stabilized liposomes: a SAXS study, Chemistry and Physics of Lipids, 165, 4 (2012) 387-392.
